# A Dignified Standard of Living in Mexico: Results of a Pilot Study of the Minimum Income Standard Approach

**DOI:** 10.1007/s11205-017-1780-4

**Published:** 2017-10-31

**Authors:** Laura Valadez-Martínez, Matt Padley, María Fernanda Torres Penagos

**Affiliations:** 1 Centre for Research in Social Policy, Loughborough University, Room U130 Brockington Building, Loughborough, Leicestershire LE11 3TU UK; 2 Escuela de Gobierno, ITESM. ITESM. Eugenio Garza Lagüera y Rufino Tamayo, Valle Oriente, 66269 San Pedro Garza García, NL Mexico

**Keywords:** Living standards, Consensual budgets, Mexico, Latin America

## Abstract

This paper explores the ways in which consensually-defined, socially-constructed living standards could be helpful in providing new ways of understanding living standards in Mexico. A pilot study formulating a “Minimum Income Standard”, carried out in the country in 2016, asked members of Mexican society what they consider to be necessary to achieve a dignified standard of living in urban Mexico today. Participants discussed the meaning of a dignified standard of living and translated such definition into concrete items in a hypothetical living room and in leisure time. Our study reveals that social participation, security, and employment are seen as important elements to live a dignified standard of living, which permeate the rationale for identifying the goods and services seen as needed to achieve a dignified living standard. The definition of a dignified standard of living could usefully inform and contribute to the ongoing debate on wage adequacy in the country.

## Introduction

Mexico has developed ways to assess living standards through the creation of poverty lines (cf. CTMP 2002) and more recently, multidimensional poverty measures (Alkire and Santos 2013; CONEVAL 2009). However, these measures have focused predominantly on objective poverty and there is no history of systematically consulting citizens within Mexico in order to define a dignified standard of living, a threshold that includes goods and services beyond basic material needs and which would enable people to be part of society. In this light, a pilot study of the Minimum Income Standard (MIS) (Davis et al. 2016) approach in Mexico asked groups of members of the public about the goods and services that people in that country considered necessary in order to achieve a dignified standard of living.

This paper explores the ways in which a socially-constructed, consensually-established living standard in Mexico could be described, based on the results of a pilot study undertaken in the summer of 2016. Research was carried out in two of the largest cities in Mexico—Monterrey and Mexico City—exploring whether people living in urban areas in Mexico could reach agreement on what constitutes a dignified ‘minimum’ standard of living—a dignified standard of living under which no one within Mexico should fall—and if so what this standard should comprise. The research builds on an established methodology, the Minimum Income Standard (MIS) approach, developed in the UK and applied in France, Ireland, Japan, Portugal and Singapore which seeks public consensus regarding the definition of minimum living standards, detailing the goods and services necessary to achieve this.

Participants in the pilot study of MIS in Mexico envisaged the following definition of a standard of living for all citizens in that country: ‘A dignified life in Mexico today is about meeting basic needs, such as food, housing and clothing, as well as having the opportunity to work, access to healthcare, education and free time. It is also about living in a stable and secure environment that allows people to be connected and be part of society’. For a country which has had around 50% of the population living in poverty during each of the last three decades (CONEVAL 2016; Székely 2005), such a definition of a dignified standard of living beyond basic material needs seems almost aspirational. Nevertheless, the MIS approach that produced this definition of a dignified standard of living presents a new way of understanding living standards in Mexico and offers the opportunity to create new indicators based on social consensus.

The MIS approach offers a novel approach in Mexico because defining and specifying living standards through public consensus allows indicators to reflect the changing nature of society and details the rationales behind why certain goods and services are necessary to achieve a dignified standard of living. The quantity and quality of items included in baskets of goods and services regarded as necessary to achieve a dignified standard of living, rooted in lived experience, could be used as a basis for assessing the adequacy of household incomes (Padley et al. 2017). Importantly, living standards derived from goods and services that people themselves have considered necessary are ‘more likely to be acceptable to society and, therefore, policy initiatives arising from them may stand a greater chance of public approval than budgets set by experts’ (Deeming 2015, p. 8). In the UK, MIS serves as the basis for setting the Living Wage, a wage floor higher than the official minimum wage (D’Arcy and Finch 2016). In Mexico, MIS has the potential to bring a fresh perspective into the debate on what is needed to achieve a dignified standard of living, for example through wages, social security or social interventions.

This remainder of the paper is structured as follows. The paper begins with an explanation of the MIS approach, which provides the conceptual framework for what is explored here. The subsequent section reflects on how the MIS approach sits within the Mexican context of measuring living standards. After that, the paper provides details about the methodology and the pilot study carried out in Mexico, while the results section examines the findings of the study in relation to what was considered to be necessary in the living room and in leisure so all citizens in Mexico would achieve a dignified standard of living. The paper concludes with an exploration of the conceptual and policy implications, and sets out critical next steps for developing the MIS approach in Mexico.

## Background

The Minimum Income Standard (MIS) approach is founded on the premise that it is possible, and indeed preferable (Viet-Wilson 1987) for minimum living standard ‘lines’ to be defined, described and agreed by the people living within any given society. That is, the definition and detailed description of what constitutes a minimum living standard should be rooted in the lived experience of individuals and households in a society rather than determined by experts, policy makers, politicians or government (Mack and Lansley 1985; Chambers 1994). This ‘consensual approach’ (Walker 1987) holds that minimum living standards are socially and culturally specific, following the view espoused in the pioneering work of Townsend (1979) that one way in which poverty can be understood is as a lack of resources *relative* to what is ordinarily or customarily seen as the approved social norm within any society. Within the MIS approach, minimum living standards are viewed as a reflection of the values in a given society. Consequently, while there may be value in attempting to identify universal or fundamental human needs (Doyal and Gough 1991; Max-Neef 1992; Nussbaum 2000), the MIS approach looks more to describe and detail first what a minimum socially acceptable standard of living is in any given society and second the ways in which this standard of living is met, by translating needs into specific goods and services, within these different settings.

The MIS approach aims to identify a minimum socially acceptable standard of living; it is a ‘minimum’ in the sense that refers to a threshold under which no one should fall; it is ‘socially acceptable’ in the sense that such a threshold is defined by society. MIS is not just about bare material necessities but about a dignified standard of living that covers all areas of life including social participation and non-material needs. In this respect, MIS refers to what a permanent standard of living should be, recognizing that while it is possible to survive on basic items, or necessities, a dignified standard of living is preferable. Consequently, the MIS approach offers a critical, and potentially alternative, perspective on what can justifiably be deemed to be a need, required in order to have a dignified living standard, within a given society. Given that MIS looks at a living standard that people consider everyone should attain, it sets a threshold that aims to reduce social inequalities.

The difference between the MIS approach and other objective indicators of well-being, such as poverty lines within any country, is that while the latter are indirect observations that do not allow for a deep interrogation and understanding of people’s needs, the former is primarily based on agreement amongst citizens of what is needed. The MIS approach asks what goods and services people consider to be indispensable for human development, many of which include subjective elements such as citizen participation or connections with others in society. As Sen states, the usefulness of wealth lies in the things that it enables us to achieve. However, this relationship is neither exclusive (since there are significant influences on our lives other than wealth) nor uniform (since the impact of wealth on our lives varies with other influences) (Sen 1999).

To establish a socially-constructed definition of a minimum socially acceptable standard of living, and a description of the goods and services needed to achieve such standard, MIS works in a qualitative way through various rounds of focus groups comprised of members of the public from a range of socio-economic backgrounds, who discuss the items that a range of different household compositions would need. The purpose of these groups is to operationalize the definition of a dignified standard of living, which was also produced by members of society, into the goods and services necessary to achieve it. People themselves define the quantity, duration, and quality of each of the items included in the list.

Different sets of groups discuss different household formations, so the detailed lists of goods and services are constructed through a process of consensus building that reflect the needs of a particular household type; for example, households with children discuss the needs of households with children and pensioners discuss the needs of pensioners. It is not unusual for those taking part in groups to start with different ideas about what is needed in order to meet the defined living standard, but consensus is reached through detailed discussion and negotiation. Importantly, where some items cannot be agreed on by a group, subsequent groups can and do resolve differences and disagreement (Davis et al. 2015). An important element of the MIS approach is that decisions from one group are carried forward to another group so ideas about the goods and services needed to achieve a dignified standard of living are iteratively constructed. A final group for each household configuration, using information and discussions from previous groups, finalizes the list of goods and services. Having various rounds of groups discussing the lists of goods and services needed to achieve a decent standard of living facilitates the formation of wider social consensus, reducing the chances of having outlier opinions.

An advantage of MIS adopting a subjective approach to define well-being—subjective in the sense that it is based on people’s perceptions and consensus reached through sharing these perceptions—is that it allows for context-specificity. MIS asks groups of people living within a particular country or setting to reach collective conclusions about what is needed by someone living in that country or context to meet both material and non-material needs. Given that the living standard based on MIS derives from conversations held in focus groups that take place in a particular time and place, the derived standard of living is sensitive to different social, economic, cultural and political environments. A minimum living standard described and detailed within Mexico is determined by those living in Mexican society and reflects the values and social norms of this society. While there may be some shared features, a minimum living standard described and detailed in Portugal, for example (Correia et al. 2016), or South Africa (Byaruhanga et al. 2017) may be very different, indicating a different set of social norms and values.

Based on the lists of goods and services agreed by focus groups, the cost per week of these items is used to calculate a weekly budget for different household types. Therefore, the description of goods and services obtained by consensus in groups results in a calculation of the income that people would need in order to reach a minimum socially acceptable standard of living defined by members of the public—a ‘dignified life’. Using the total budget that would cover the cost of the list of goods and services considered by society to be needed to achieve a dignified standard of living, MIS makes it possible to look at how many households do not have the income required to meet this standard (Davis et al. 2016; Padley et al. 2016, 2017).

## How the MIS Approach Sits Within the Mexican Context of Measuring Living Standards

### Expert-Defined Versus People-Led: Rooted in Lived Experiences

The MIS approach shares some similarities with the existing wellbeing lines in Mexico, which assess household income against a food and a non-food basket (CONEVAL 2016). The food basket is based on expert-defined nutritional requirements according to age and gender and adapted to the Mexican diet. CONEVAL’s food basket takes into consideration the work of various researchers and experts in the medical and nutrition sciences such as Bourges et al. (2005) and Muñoz de Chavez (2002), as well as the guidelines of international institutions such as the Economic Commission for Latin America and the Caribbean (ECLAC), the Food and Agriculture Organization of the United Nations (FAO), and the World Health Organisation (WHO). Previous methods for defining a food basket in Mexico, The General Coordination of the National Plan for Depressed Areas and Marginalized Groups, namely COPLAMAR in the 1980s and INEGI-CEPAL in the 1990s, were also based on medical and nutritional expert knowledge (see Avila Curiel 2011 for a review).

The non-food basket in Mexico, used in the official multi-dimensional poverty measurement, is based both on consumption and on perception of need, captured through a survey developed by Hernandez-Laos (as cited in CONEVAL 2012). The information for the non-food basket was obtained through two surveys: The National Survey for Income and Expenditures (ENIGH) for actual consumption and a specially-designed survey for perceived needs. The non-food basket followed four criteria to evaluate which goods would be included: goods regarded as necessary according to economic theory (those with an income elasticity lower than one, meaning that demand for such goods does not change much relatively to change in prices); goods that at least half of the population indicated were necessary; that overall expenditure on these goods in the country is above average consumption for all goods; and that at least 20% of households in the reference group consume that good (Cantú et al. 2004). The non-food basket in Mexico includes 261 items for rural and 269 items for urban settings within the following categories: public transport, household maintenance, personal care, education, culture and recreation, communications, housing costs, clothing and footwear, household goods, healthcare, and leisure (CONEVAL 2012). Looking specifically at the areas considered in the pilot study, the living room and leisure, the official non-food basket in Mexico includes curtains, bookcases, television and television stand, radio, computer, CD player, books and encyclopedia, newspapers, magazines, audio cassettes, and compact discs.

The official non-food basket in Mexico is the most accurate calculation of the goods that society in that country has considered to be necessary. However, there are two limitations to the official non-food basket. Firstly, it is based on a relatively narrow definition of social participation, including only a small number of items classified under leisure (books, encyclopedia, newspapers, magazines, audio cassettes or compact discs) and a modest budget for parties but no budget for other regular activities like hobbies, sports, or socialization outside the house. Secondly, public views on what items are considered as necessary (those which at least 50% of the survey agreed as necessary) are adjusted based on actual consumption, and items are included in the final basket only if their consumption is higher than the mean of all items listed and if they are consumed by at least 20% of the reference population (CONEVAL 2012). As some scholars have pointed out (Brewer and O’Dea 2012; Thorbecke 2005), using consumption as a way to measure living standards could be problematic because people do not necessarily consume what is needed, often due to income restrictions. In addition, the consumption of market products is an incomplete indicator of wellbeing taking into account that there are goods and services whose market price may not be available or may have distortions (Alkire and Foster 2011; Bourguignon and Chakravarty 2003).

Another difference between the MIS approach and the way in which existing wellbeing lines within Mexico are defined, is that while wellbeing lines are specified by experts at various academic, governmental, and international institutions, the MIS approach is based on what members of society agree is needed. In this sense, MIS offers a bottom-up rather than top-down approach, where members of the public define, discuss and agree what constitutes a minimum acceptable standard of living in Mexico as well as describing the items that would be required to achieve this. Rooted in lived experience, the MIS approach affords the opportunity for the public to construct a detailed description of what a decent standard of living in Mexico should be, thinking beyond the most basic material requirements of living, not bound by current income constraints.

The fact that MIS is based on the consensual agreement of members of the public empowers citizens, involving them in the creation of new social indicators and enabling them to reflect on and define the goods and services needed to achieve a dignified standard of living. As Holguín (2013) mentions, it is necessary to opt for a more active participation ‘that allows the citizens different institutional arrangements with public and private organizations around programs that respond to their own realities’ (p. 190). Conceptually, the MIS method has the potential to contribute to the understanding of living standards by providing people with the opportunity not only to indicate whether an item is needed in a minimum basket of goods but also to discuss what quality this item needs to be and to outline the rationale/s for including it. As a result, this method could enrich the conceptual definition of living standards in Mexico, by allowing society to detail the goods and services that would be needed to satisfy such a standard.

### Updating the Baskets: More than Inflation

Another important potential contribution of the MIS approach to understanding living standards in Mexico is related to how the baskets of goods and services needed to live a decent standard of living are updated. The wellbeing thresholds currently used by the Mexican government have only been updated with inflation (CONEVAL 2016), the contents of the official baskets in that country have been maintained since it was first established and, according to official documents (CONEVAL 2012) the plans to revise the lists are limited to once every 10 years. A significant element of MIS, and qualitative research on living standards in general, is that it helps to capture societal changes, patterns of consumption, and preferences regarding the elements necessary to achieve a dignified standard of living. MIS allows the opportunity to revisit and ‘rebase’ the items included in the baskets, based on what society agrees as necessary to live with dignity in a particular context, at a particular time. The discussion groups at the heart of the MIS method consider in detail the items in a basket of goods and services that the public agree is needed in order to achieve a decent standard of living, so any additions or changes are reflected on and supported through clear rationales (cf. Davis et al. 2015).

In the United Kingdom, baskets of goods and services included in the MIS project are updated every 2 years (Davis et al. 2016). In the UK, for example, households above pension age introduced a home computer and internet access into their budgets in 2014, arguing that being able to access online facilities was necessary for personal development and in order to be part of society (Davis et al. 2014). Currently, the non-food basket in Mexico is updated only with inflation (CONEVAL 2012) and expanding the pilot of the MIS project could provide the opportunity to change or adjust the contents both in terms of quantity and quality as well as price. At this point, the pilot in Mexico is only exploratory, but the MIS approach has the potential to provide valuable insight into how societal expectations about need could, or perhaps should, shape the basket of goods and services needed to live a decent standard of living.

## Methodology

The research on which this paper is based was carried out in Mexico in the summer of 2016. It consisted of six focus groups, three in Mexico City and three Monterrey, which were selected as the sites for the research as their populations made it possible to capture a broad representation of urban Mexican society. This exploratory study concentrated on families with children because they represent around 90% of the Mexican population (INEGI 2017).

Following the MIS approach, each of the focus groups comprised individuals from a range of socio-economic backgrounds, including people from across manual and professional occupations, with various levels of education and household incomes across the distribution, from a variety of housing types. This diversity is critical as the MIS approach aims to develop consensus within economically and socially mixed groups, helping to ensure that consensus about living standards is inclusive rather than simply representing the preferences of a particular section of the income distribution (Davis et al. 2015; Deeming 2015). Each of the six focus groups included ten individuals (with a mix of genders in each group, at least four men in each case) who were working age parents (aged 19–55) with children aged between 0 and 16 years. Participants of the focus groups were recruited by specialized agencies in Mexico City and in Monterrey, purposely filling quotas for gender, five income brackets, and six occupational ranks to ensure representation of various socio-economic backgrounds in each of the six focus groups. Recruiters received training from the research team on the MIS approach and the specification of participants needed for the pilot study.

The first two groups (one in Monterrey and one in Mexico City) were responsible for constructing a definition of a dignified standard of living in Mexico. These groups discussed in detail the elements that combine to describe a dignified standard of living in Mexico, the definition presented at the beginning of this paper. The purpose of these first two ‘orientation’ groups (see Davis et al. 2015) was to explore what a minimum socially acceptable standard of living means and whether or not groups were able to reach agreement about how this minimum living standard should be described within Mexico. Given that the MIS approach asks groups not to think of their own preferences or needs, but rather to think about those of hypothetical individuals, the first two groups were also responsible for developing and describing two case studies to be used within subsequent groups: one comprised two parents and two school-aged children, and one comprised a single parent with two school-aged children. Case studies provide advantages against referring to the individual preferences of those in the group; they help participants to not feel intimidated or judged about their choices and opinions, and they help to construct a single vision based on consensus instead of reflecting the particular needs and preferences of each individual in the group (see Merriam 1998; Stake 1994). The case studies developed by the first two groups were used in each of the remaining groups to stimulate discussion about what two different types of households in Mexico would need to in order to have a dignified standard of living.

Following on from the first two groups, four more groups were undertaken, two each in Mexico City and Monterrey. These groups worked on translating the definition of the ‘dignified life’ drafted by the first two groups into the goods and services needed to achieve such a standard of living. The groups reached agreement on the quantity and quality of items in a hypothetical living room as well as discussing the frequency and type of leisure activities that the case study households would need to be able to do in order to achieve the described standard of living. Importantly, groups deliberated and provided reasons why the amount and quality required for each element, goods or services, was needed to achieve this living standard. Given that one of the purposes of the pilot study was to explore whether people from very different socio-economic backgrounds could reach consensus on a standard of living that could be universal for urban Mexico, the discussions in the groups concentrated on the living area and on leisure because these are areas of life that could potentially provide sufficient level of agreement but also a significant level of debate, based on experiences from previous MIS studies in other countries.

Each of the groups were moderated by the same two researchers in order to facilitate continuity in the discourse and decisions from one focus group to another. Discussions were structured through the use of a ‘topic guide’ similar to the ones used in MIS projects in other countries to enable consistency in approach across different contexts. Each of the six focus groups lasted 2 h and groups were conducted in Spanish to ensure that implied meanings in the discussions around living standards were captured.

## Results

The definition of a dignified standard of living that resulted from the first two groups, presented at the beginning of this paper, was reviewed by the four subsequent groups to explore the extent to which there was agreement about this across groups. This same definition was used throughout to identify the elements that the case study households would need in order to achieve this standard; pilot study was relatively narrow in scope, concentrating on items in the living room and on leisure. In all groups, participants were clear that a dignified standard of living referred to a condition beyond survival and made a distinction between ‘living with dignity’ and ‘basic material needs’.

### Setting the Stage: What Constitutes a Dignified Life

People in the groups undertaken in Mexico agreed that a dignified standard of living should include food, clothing, housing, public services, transport, access to healthcare, access to education, and leisure time, as well as employment opportunities. The first item that groups operationalized in relation to this definition was housing. All participants of the pilot study in Mexico were clear that as a minimum, housing in urban Mexico should be constructed from durable materials in its entirety (walls, floor, and roof made of cement or brick), and that all houses should have basic services. The rationale for having durable materials as a minimum was that ‘such material is more resistant to the climate (in Mexico)… it gives security. If there is a hurricane or something, all that you have worked to achieve will not be gone’ (Female, Monterrey, Group 1).

Another characteristic of housing expressed as needed by members of all groups in Mexico was the need to have separate areas within the house. Participants in the groups agreed strongly that, in order to live with dignity, housing should have distinct areas for sleeping, cooking, bathing, doing laundry and socializing. The arguments for having these separate areas were linked to privacy, hygiene, security, as well as physical and mental health. For example, it was stated that the living area was an area to socialize with friends and having a separate area for sleeping was needed ‘for privacy, if your children have a get together and you want to be in pajamas’ (Female, Monterrey, Group 1).

A central finding of this pilot study is that the overall conception and description of what constitutes a dignified standard of living was shared across all groups. Using this definition of a dignified standard of living in Mexico, there was agreement that such a standard entails having a house with a living area that enables people to relax and socialize, as well as including a budget to take part in leisure activities outside the house.

### Living Room: Sitting and Sharing

The items in the living room that participants identified and discussed as necessary to achieve a dignified standard of living include some that serve a practical purpose and some that also serve a social function. In the first category, groups consistently talked about including elements to satisfy practical needs of storage and lighting. Groups agreed that it was necessary to have a bookcase to keep the living area tidy, a lamp (in addition to the central light fitting) to help with eye health, and curtains to protect the area from direct sunlight and also provide safety and privacy. The quality and duration of these goods were discussed, and there was clear consensus that these should not be the cheapest version available because they would not provide value for money and/or could be made of materials that could be risky for children, but also that they did not need the most expensive ones. In order to meet a minimum dignified standard of living, it was agreed by groups that these items would need to be of low-to-mid quality, with each lasting 10 years.

One element that also serves a practical purpose but where there was a clear geographical variation within Mexico is a fan. This was considered to be a necessity in Monterrey, which has a semi-desert climate reaching very high temperatures in the summer, but not in Mexico City, which has a mild climate throughout the year. Groups in Monterrey were clear that while a fan would be sufficient to satisfy the need of maintaining good health, air conditioning would be a luxury, and therefore should not be included.

Some items in the living area would serve a dual function, being both practical and satisfying the need for social participation. For example, groups included comfortable seating for family and guests that would be of mid-low quality and would last 7 years. Participants argued that sofas would serve the purpose of providing somewhere to sit, citing health and comfort reasons, as illustrated in the following discussion:

Moderator: Why is it important to have a place to sit?

Female 1: To be comfortable.

Male 1: To be able to rest.

Female 2: To talk to others at ease. To watch TV… To spend time with others…

Female 3:… with family

Female 4:… with friends, with everyone

(Monterrey, Group 5)

Across all groups, participants agreed that sofas also help to fulfill the needs of social participation, allowing family members ‘to have the right to invite friends over… because it is part of a dignified life to have the opportunity to share with others, to laugh, to enjoy’ (Male, Mexico City, Group 3). All groups also agreed that family were likely to come over to one’s house unannounced, which is customary in the country: ‘if one or two visitors turn up, your father-in-law or your mother-in-law, then you have somewhere to invite them to take a seat’ (Female Mexico City, Group 4); and it is important to have visitors over ‘to spend time together … for family unity … to be part of society’ (Mexico City, Group 4). The conversations held in groups with regard to spending time with family echo the findings of other research that highlights the importance of family ties in Mexican culture (García-Coll and Vázquez García 1995; Sabogal et al. 1987), which has ‘a strong sense of familism that implies that family members should be close to one another and support one another’ (Hardway and Fuligni 2006, p. 1246).

Another item in this category serving more than one purpose or function is a television. Participants said that a 32 inch television would satisfy the need of keeping oneself informed and updated about issues like weather, and political or economic news, while also providing a source of entertainment, ‘… and recreation is part of a dignified life’ (Female, Mexico City, Group 3). Even though some participants recognized that watching too much television could contribute to isolation and health problems, there was clear consensus that a television is needed to achieve a dignified standard of living because it would help to fulfill the need for social interaction. It was clear from groups that watching television with other family members or friends was a social norm. The following discussion around why a television would help the case study to achieve the standard of living described in the definition illustrates this point:

Moderator: Why is it important to have a television (to have a dignified standard of living)?

Female 1: It provides entertainment… to entertain the family because it provides leisure but also helps you bond together.

Female 2: (It provides) information, isn’t it?

Male 1: To spend time together.

Female 3: Yes, to spend time together.

Female 4: To distract oneself, to forget about everything for a little while.

(Mexico City, Group 4)

Groups agreed that the television would need to be a ‘smart TV’ given that a change in technology in January 2016 (SCT 2016) forced Mexican households to switch from an analogue to a digital signal. Even though research on ‘social television’—in terms of interaction on social media while watching television—is relatively new (Lin et al. 2016), research regarding television as a form of social interaction has a longer history (Levy 1978; Rubin et al. 1985). This research has found that watching television with others and watching the same program as others are each associated with feelings of belonging and the need for company (Cohen and Lancaster 2014), which were implied in the conversations held in groups in Mexico. Similarly, participants in MIS research in other countries have agreed that having a television helps to fulfill the need for information, connectedness, and social interaction (see Collins et al. 2012 for Ireland and Davis et al. 2016 for the UK).

Following from discussions about the purpose and function of a television, groups also discussed the inclusion of a subscription video streaming service such as Netflix under the rationale that households would have a smart TV and an internet connection anyway. Those in favor of including Netflix as something that is needed to meet a dignified standard of living argued that it provided a wider variety of entertainment than ‘normal TV’ and that the opportunity to access content would allow people to feel integrated in society and to have something to talk about with others. Conversely, participants who argued against including Netflix as a necessity, stated that those purposes could be satisfied with ‘normal TV’ and that there was no need to pay for these extra services. This issue was discussed and debated at length in all four groups who were tasked with building the lists and the final decision was that paying for streaming TV goes beyond the minimum needed to live a decent standard of living.

With regard to access to the internet, there was consensus that having an internet connection in the household is needed ‘to live a dignified life today’ (Male, Monterrey, Group 6). Virtually all participants agreed that having access to the internet is necessary in today’s society in order to enable access to information, as a tool for children to be able to do homework, for accessing employment opportunities, and for undertaking tasks such as paying bills or looking for information on crime. The issue of using the internet for safety reasons came out consistently across the pilot groups; participants highlighted the importance of being able to stay in contact with children when they go out or using the internet to check if there are problems like a shoot-out in any part of the city. Research in Mexico has found that given the high risk that journalists in traditional media have been facing in the last few years, social media has become more prominent as a source of information on security risks (Gonzalez de Bustamante and Relly 2014): ‘Current technological advances have allowed for the use of the Internet as a safe haven to broadcast news related to drug trafficking activities from various regions of the country in an uncensored, unrestricted manner through the use of social networks’ (Correa-Cabrera and Nava 2011, NA).

Access to technology has also been found in the literature as associated with inequality; ‘technological progress rather than trade has been the mechanism through which the unequalizing effects have been operating’ (Behrman et al. 2003, p. 26) across Latin America. Even though technology has the potential to encourage and enable progress, including industrial development, access to the internet has also been regarded as a tool for income generation in developing countries (Kenny 2002). The relationship between access to technology and the structural inequalities in the country was something that was raised by participants, as illustrated in the words of one of the participants when discussing the definition of a dignified life:

I would add access to technology but freely available. (…) For example, when there was the Formula 1 event in Mexico, the children who sang the national anthem were given a tablet (as a way to say thank you) but then they got to their communities and try to explain to them that it would not work because there was no internet (Male, Monterrey, Group 1).

An item that was regarded by groups as closely related to entertainment and social inclusion but with little practical purpose was a videogame console, an item on which there were a range of opinions. Participants who considered this to be a necessity in order to achieve a decent standard of living indicated that it would provide leisure and ‘the opportunity to be in touch with people in other countries’ (Female, Mexico City, Group 3) who are playing the same game. Further, some argued that it provided entertainment for children in a safe environment and that playing inside the house is better than outside because it gives ‘peace of mind to the parents; children are entertained for hours and hours’ (Female, Mexico City, Group 3), referring to safety issues, particularly given the crime rates in Mexico. Other arguments included that children need to play similar games to their friends, because otherwise ‘they would feel rejected, isolated’ (Male, Mexico City, Group 4). Similar to the arguments against Netflix, those who were against including a videogame console in the list of items needed to have a decent standard of living stated that there were other options to obtain leisure and entertainment inside the house, such as board games or going to the park. The final decision reached by groups was not to include a videogame console in the list.

Another issue that prompted debate amongst participants was interior decoration, which for many in groups did not serve a practical purpose and would therefore not be classified as necessary for achieving a decent standard of living. However for other participants in groups having a decorated living area was seen as being necessary to achieve a dignified life because decorative elements give comfort, peace of mind, warmth, happiness (Mexico City, Group 3), ‘family ties’ (Female, Mexico City, Group 4) and can help people ‘to feel connected, to feel part of society… you need to arrive to a homely place, that gives you a sense of belonging, that you like it when you arrive… to have a pretty house, with decoration, where you feel like you want to be there’ (Male, Monterrey, Group 5). Research elsewhere has found that personalization of one’s house is associated with self-identity and with shaping social relations (Fidzani and Read 2014; Hirschman and Belk 2014) and that elements used to personalize a living space could have social meaning (association with others), memory meaning (reminder of a person, place, or moment), and self-meaning (representation of oneself), apart from the functional properties and the enjoyment that decorative items may bring (Kamptner 1995).

In this study, being able to decorate the living area was also linked to feeling part of society because it would provide the ability to display pictures of family members or cultural references and because it is a space in which to spend time with family and friends:

Female 1: It gives you warmth, harmony; you feel united.

Male 1: Unity.

Female 1: It is very important; I think the living area is the most important area.

Male 1: Yes, because you are with your son, your wife, with friends. (Decoration is important) because it welcomes you, it makes you feel fine.

(Mexico City, Group 3)

Those in favor of including some decorative items indicated that mirrors, vases, portraits, framed photographs or similar elements improve the emotional wellbeing of people, and help families ‘to have a sense of belonging’ (Male, Monterrey, Group 5). Nevertheless, other members of the groups reminded fellow participants that their task was to find a standard of living that would allow anyone in Mexican society to live beyond survival and that personalizing one’s home is a way to feel better about oneself (e.g. diplomas or sports awards) and to show some form of connection with loved ones (e.g. family photographs). The final consensus was that a budget should be included to purchase items that could reflect individual choices and preferences, not necessarily a specific list of decorative items. The decision was to include a modest budget of 1000 Mexican Pesos per year (45 USD), so households could decide how to spend it.

### Leisure: Beyond Material Needs

The second element or domain discussed in this pilot study was leisure, which was articulated in the definition of a dignified life that came out of the orientation groups, as having free time. Participants were clear that social participation is a crucial part of a dignified standard of living; ‘you cannot only live to work; neither only to study. You need some leisure time to detach from the stress that (work) entails and to strengthen family ties’ (Female, Monterrey, Group 1). Similarly, MIS research carried out in other countries has found that the public agree that a decent standard of living includes opportunities to participate in society (Correia et al. 2016; Davis et al. 2016).

When translating what is needed in relation to leisure into specific items, two issues are evident from the discussions in Mexico. Firstly, most recreational activities are assumed to be undertaken as a family, rather than as individuals or independently, and secondly holidays are agreed to be an important aspect of living with dignity. When describing the type of leisure activities that are required to live a dignified life, participants talked mainly about going to the movies, eating out, and going to the park, all done as a family; ‘weekends always in family’ (Female, Mexico City, Group 4). Groups recognized that there are many free options available that would enable families to spend quality time with each other; for example, ‘going out with your child to the park, spending time together, having quality time’ (Male, Monterrey, Group 6). However, there was agreement that living with dignity implies not having to be limited to doing free activities and that people should be able to go out, paying for some activities that afforded ‘the opportunity to spend time with your family, to clear your mind, and to be relaxed before starting a new week’ (Male, Monterrey, Group 5). The final decision of groups in Mexico was to include a budget for activities that would allow families to fulfill their need for social participation. Participants agreed that using a model built on going out to the movies and having a family meal in an inexpensive restaurant would need a budget of 1500 Mexican Pesos per family (70 USD), twice a month.

Secondly, all groups in their discussions of free time as part of a dignified standard of living, mentioned the need to have some holidays away from home. The reasons given for including holidays as part of a decent standard of living included: physical health, mental health, rest and de-stressing, a change in routine, spending time with family, building memories, having a good quality of life, learning and embracing culture. All groups agreed that as a minimum, families with children in Mexico need a 1-week holiday each year to a destination within the country. The legal period of annual leave in Mexico is between 6 and 10 days per year in the first 3 years of employment^1^ (LFT 1970) and official figures estimate that the Mexican population go on holiday for 6 days on average (SECTUR 2013). Given these legal limitations on annual leave, it is understandable that all groups agreed a 1-week holiday and not a longer duration, taking also into consideration distances in Mexico, assuming transport by road: ‘it takes one day just to travel; it takes 24 h just to get where you want to go’ (Male, Monterrey, Group 6).

When defining the details of the annual holiday, groups were clear that the budget would need to cover the cost of an all-inclusive four-star hotel. When asked why it would need to be a four-star hotel, there was strong consensus that other options such as a self-catering apartment would cost a similar amount and that a hotel of this standard provides a level of hygiene, security, and safety that other options do not. The issue of security has clearly influenced what is needed in order to meet a minimum dignified standard of living, as illustrated in this conversation:

Female 1: For a decent standard of living, it’s a hotel.

Male 1: A hotel.

Moderator: What about one, two, three stars for a dignified standard of living?

Female 2: Three.

Male 1: Four! In a three-star hotel there is no safety; the four stars one provides safety.

Female 3: If you go to a three stars… there are cockroaches!

Female 4: The four stars one is safe.

Male 2: It also gives you peace of mind.

(Mexico City, Group 4)

### Structural Issues Around Living Standards: Work as a Fundamental Factor

Conducting groups using the MIS methodology allowed us to explore questions concerning structural issues around wellbeing and the practicalities of achieving a decent standard of living in Mexico. A clear theme that came out of the groups was the central importance of work. On the one hand, work was described as a means of personal development ‘because it gives you stability; work is always linked to emotional stability’ (Female, Mexico City, Group 2); ‘life is about being happy and work is also part of that’ (Male, Mexico City, Group 4) and a way of ‘personal development. It is a step in your life, and in one way or another you are going a step up’ (Male, Mexico City, Group 2). It is interesting to note that work has been identified as an element of wellbeing, but is not included in the dimensions of the official poverty measurement.

On the other hand, work was consistently regarded as ‘the pillar to obtain everything else. I think work is the pillar, because everything else is derived from there; education, health services, food, everything’ (Female, Mexico City, Group 2). This view indicates a broader Mexican context, where safety nets are ‘conspicuously absent or ill-designed and insufficient’ (Gasparini and Lustig 2011, p. 2) and where social policies have mainly been narrowly targeted, oriented primarily to those living in poverty. Government actions aimed at increasing people’s economic wellbeing principally take the form of cash transfers or monetary aids to specific groups, for example the recently launched Program for the Promotion of Social Economy (*Programa de Fomento a la Economía Social*) which provides a monetary aid for those whose income is below the poverty line and who would like to start a small business.

In Mexico, ‘work is where most of household income is generated as well as the inequalities related to its distribution’ (CEPAL 2014, p. 140). It is well known in the literature (Levy 2008) that one of the main problems in Mexico is informal labor. It is estimated that about 60% of people who work do so informally (Ortega Díaz 2013), meaning lower tax collection for the state and lack of social benefits such as pensions or health insurance for those who are working informally. Even for those working in the formal labor market, there are no income protection measures related to unemployment—like schemes found in European countries. Despite the creation of an unemployment benefit, which the Mexican Congress approved in 2013, and which was due to start at the beginning of 2016, this has not yet been implemented (El Economista 2016). This situation is illustrated in the words of one of the participants: ‘If there is no work, there is no income, and you don’t have anything else’ (Female, Mexico City, Group 2). Consistently, there was awareness that in Mexico, virtually the only way to achieve a dignified standard of living was through work-related income:

Female 1: I consider that we have to know the income of the family to know what is decent.

Male 1: Translated into a commercial society that is the one we live in, what is decent is what you can afford. All this translates into money and it’s a lot of money.

Female 2: Unfortunately, dignity does have a price.

(Mexico City, Group 3)

Given the narrowness of income protection policies in Mexico, people living in poverty ‘do any kind of thing to survive’ (Tello 2010, p. 22). In this research, there was a generalized assumption that work is the best, if not the only, way to achieve a decent standard of living in Mexico. Further, participants were in clear agreement that, even if work is secured, wages are not sufficient to obtain the standard of living that they were describing:

a lot of the times, you have a job and are working for eight hours (a day) … and your salary should be enough to achieve a decent living standard. But in Mexico that is not possible… I know a lot of people who are working with all their effort but it is not enough, and then you have to sell something on the side, sell shoes or something (Female, Mexico City, Group 2).

Existing evidence indicates that real minimum wages in Mexico have declined almost 75% since the late 1970s, with the worst annual figures during the 1980s (− 6.4% annually) and with little or no change after 2000 (Escobar Toledo 2014). The inadequacy of wages was a topic that consistently emerged in groups. All emphasized that despite the fact that work is the way to achieve a dignified life in Mexico, wages and particularly the minimum wage, do not help families to meet the standard of living that groups had agreed to be the minimum necessary to live with dignity, ‘the minimum wage does not allow you to have a dignified life’ (Female, Monterrey, Group 1).

Looking at wage figures since 2005, evidence indicates that the minimum wage has only covered between 77 and 81% of a person’s food basket for those living in urban areas (Escobar Toledo 2014). As one would expect, if the minimum wage is not sufficient to acquire a food basket, it is way below the budget that would be needed to purchase the goods and services that constitute a decent standard of living, as shown in the following discussion:

Male 1: A lot of people get stuck and cannot provide their children with the education that they would like to, so if children reach certain age and cannot study, then they have to work and that is a chain for many people.

Male 2: In fact, the minimum wage is not even enough for public (education).^2^

(Monterrey, Group 1)

This discussion on the (in)adequacy of wages in Mexico is an area in which the MIS approach could potentially make a significant contribution to policymaking. One of the criticisms that has been made of existing ways of defining wages, not only in Mexico but globally, is that ‘the specific method by which human needs are incorporated into the process of determining minimum wages and poverty levels is often not transparent’ (Shelburne 1999, p. 3). In Mexico, the debate on wage adequacy has regained prominence in the last few years among scholars and policymakers. For example, the director of the National Commission for Evaluation, the body responsible for measuring poverty and evaluating social policy in Mexico, recently argued that increasing the income of families is the most relevant element in poverty reduction (as cited in FORBES 2016). The National Commission for Minimum Wages in Mexico states ‘the minimum wage should be sufficient to satisfy the normal needs of a family head in material, social and cultural aspects as well as to provide for the compulsory education of children’ (CONASAMI 2016). Nevertheless, with more than 13% of the economically active population earning the minimum wage and around 65% of the active population earning at most three times the minimum wage (INEGI 2015), this intention is still far from reality and a clear sign that the minimum wage should be reconsidered.

## Conclusions

This paper has presented explored the results of a pilot study of the Minimum Income Standard (MIS) approach in Mexico, with the aim of finding new ways to understand living standards in Mexico. Six focus groups carried out in Monterrey and Mexico started by discussing what it means to have a dignified standard of living. The definition of a dignified standard of living in Mexico that resulted from the pilot study, not only takes into account basic needs as food, housing and clothing, but also having the opportunity to work, access to healthcare, education, and leisure time. Further, in the Mexican context, a dignified life involves living in a stable and secure environment that allows people to be connected and to be part of society. Groups in the pilot study also translated that definition of a dignified standard of living into specific goods and services inside and outside the home. More specifically, participants in the pilot study provided the quantity and quality of items that would be necessary in the living room of a hypothetical, case study family in urban Mexico as well as part of leisure, explaining the reasons why each of those are needed to achieve such a standard.

Given that the main purpose of the pilot study was to explore whether it would be possible to reach consensus in a highly unequal society and the discussion concentrated only on the living room and on leisure time, there are some limitations on the extent to which it is possible to compare the goods and services agreed to be necessary to achieve a dignified standard of living—based on the MIS approach—and other baskets like the one used by official measures. Nevertheless, even when looking only at these two components, the living room and leisure time, important differences emerge between the consensual approach and objective observations. While the non-food basket used in official measurements of poverty in the country includes items that were also mentioned in the pilot study of MIS in Mexico as part of the living room (e.g. bookcase, table, television, computer, curtains, books, audio equipment), participants in the pilot suggested that other items were also needed to achieve a dignified standard of living. For example, participants in the pilot agreed that households with children in Mexico should be able to afford some decorative items. According to participants, decoration satisfies the need of feeling connected with society and helps feeling well with oneself, both of which are important to achieve a dignified standard of living. With regard to leisure, the official non-food basket includes a budget for parties (cost of renting a venue, food, and entertainment), but participants in the pilot of MIS in Mexico agreed that in order to achieve a dignified standard of living, it is important to include a budget for regular social activities outside the house, like pursuing hobbies, going to the cinema or eating out with the family.

The pilot was successful in finding that consensus could be reached, in terms of the discussion enabling people to reach a jointly formulated view of a standard that everyone in Mexico needs and should be able to have. The process of reaching consensus was successful in all groups, despite the fact that at first it appeared challenging for some participants to think beyond what one could afford and engage with the task of defining the items that a typical family with children in Mexico would need to live with dignity. For many participants, it was complicated to think of a dignified standard of living that is above basic survival, but within groups participants also noted that the list of items that had been identified as necessary to reach a dignified standard of living ‘are basic things that we all should have; we all could have that but given the circumstances of the country and the government, we do not have that’ (Male, Monterrey, Group 5).

Practically, the MIS methodology is an advance in identifying people’s needs and has the potential to become an important tool for the development of public policy. In the pilot study, members of the public have identified structural issues that are constraining living standards in Mexico, with one structural issue consistently arising in conversations, namely the adequacy of work and wages. In policy terms, the definition of a standard of living based on people’s lived experiences and rationales could inform the debate on wage adequacy, and open the possibility to debate other forms of social protection that are not necessarily linked to income received through employment.

A by-product of the pilot study is the potential to empower citizens through their reflections on their own needs and constraints. In the MIS research in Mexico, it was evident that there is generalized awareness of what should constitute a decent standard of living but that this is an aspiration more than a reality for many within Mexican society. Groups showed a strong sense that, as Mexico is a country where many aspects of life are commodified, income coming from employment seems to be the most important way to obtain a decent standard of living.

The pilot study of the MIS Project carried out in Mexico throws light on living standards in the country, as consensually defined by members of the public. An important aspect of MIS in Mexico is that it could foster the creation of new social indicators around a dignified standard of living; according to participants in the groups of the pilot study of MIS in Mexico, basic material needs are not enough. A dignified standard of living also entails elements that would allow people to feel part of society. Social connectedness is a crucial component of a dignified standard of living, an issue that emerged from the pilot and which has emerged in other countries where the MIS approach has been carried out, bringing to the fore the potential to expand current conceptualizations of living standards to include a broader understanding of social participation. Expanding the pilot to include other household types and all areas of household expenditure would be the clearest next step.

## Footnotes

1 By law, 2 days of leave are granted for each year of service until the fourth year of service; 2 years are then added for each 5 years of service. Employers could choose to give out more than the legal requirements of leave, but the vast majority adhere to the period set out by law.

2 Public education is significantly less costly than private education in Mexico.
